# A genome-wide linkage study of autism spectrum disorder and the broad autism phenotype in extended pedigrees

**DOI:** 10.1186/s11689-018-9238-9

**Published:** 2018-06-11

**Authors:** Marc Woodbury-Smith, Andrew D. Paterson, Irene O’Connor, Mehdi Zarrei, Ryan K. C. Yuen, Jennifer L Howe, Ann Thompson, Morgan Parlier, Bridget Fernandez, Joseph Piven, Stephen W. Scherer, Veronica Vieland, Peter Szatmari

**Affiliations:** 1 Institute of Neuroscience, Newcastle University, Sir James Spence Institute, Royal Victoria Infirmary, Queen Victoria Road, Newcastle upon Tyne, NE1 4LP UK; 2 Program in Genetics and Genome Biology, The Centre for Applied Genomics, The Hospital for Sick Children, Toronto, ON Canada; 3 Division of Epidemiology and Biostatistics, Dalla Lana School of Public Health, University of Toronto, Toronto, ON Canada; 4 Department of Psychiatry and Behavioural Neurosciences, McMaster University, Hamilton, ON Canada; 5 Carolina Institute for Developmental Disabilities, University of North Carolina at Chapel Hill, School of Medicine, Chapel Hill, NC USA; 6 Provincial Medical Genetics Program, Health Sciences Centre, St. John’s, Newfoundland Canada; 7 McLaughlin Centre and Department of Molecular Genetics, University of Toronto, Toronto, ON Canada; 8 Battelle Center for Mathematical Medicine, The Research Institute at Nationwide Children’s Hospital, Columbus, OH USA; 9 Centre for Addiction and Mental Health, The Hospital for Sick Children and University of Toronto, Toronto, ON Canada

**Keywords:** Autism spectrum disorder (ASD), Genome-wide linkage, Posterior probability of linkage (PPL), Family genetics, Extended pedigrees

## Abstract

**Background:**

Although several genetic variants for autism spectrum disorder (ASD) have now been identified, these largely occur sporadically or are de novo. Much less progress has been made in identifying inherited variants, even though the disorder itself is familial in the majority of cases. The objective of this study was to identify chromosomal regions that harbor inherited variants increasing the risk for ASD using an approach that examined both ASD and the broad autism phenotype (BAP) among a unique sample of extended pedigrees.

**Methods:**

ASD and BAP were assessed using standardized tools in 28 pedigrees from Canada and the USA, each with at least three ASD-diagnosed individuals from two nuclear families. Genome-wide linkage analysis was performed using the posterior probability of linkage (PPL) statistic, a quasi-Bayesian method that provides strength of evidence for or against linkage in an essentially model-free manner, with outcomes on the probability scale.

**Results:**

The results confirm appreciable interfamilial heterogeneity as well as a high level of intrafamilial heterogeneity. Both ASD and combined ASD/BAP specific loci are apparent.

**Conclusions:**

Inclusion of subclinical phenotypes such as BAP should be more widely employed in genetic studies of ASD as a way of identifying inherited genetic variants for the disorder. Moreover, the results underscore the need for approaches to identifying genetic risk factors in extended pedigrees that are robust to high levels of inter/intrafamilial locus and allelic heterogeneity.

**Electronic supplementary material:**

The online version of this article (10.1186/s11689-018-9238-9) contains supplementary material, which is available to authorized users.

## Background

Autism spectrum disorder (ASD) is a neurodevelopmental disorder defined by characteristic social deficits and ritualistic, repetitive behaviors with onset in early childhood. Recent prospective data from the Baby Sibs Consortium show roughly one in five younger sibs of an older affected proband are themselves affected [1], much higher than population prevalence rates of roughly 1% [2] indicating familial clustering. A recent meta-analysis of twin studies demonstrates that the basis of this familiality is genetic; concordance for autism or for a phenotype that includes milder cognitive and social communication deficits and rigidity (termed the broad autism phenotype or BAP) was > 90% among monozygotic twins, compared with ~ 10% in DZ twin pairs [3]. The prevalence of BAP itself is between 14 and 23% of parents of children with ASD and 5–9% of comparison parents [4].

It has been known for a long time that ASD is common in individuals with certain genetic syndromes, either by chance or by shared genetic susceptibility. Recent reviews describe over 100 genetic loci associated with ASD [5, 6]. This may represent a chance association or a causal relationship, although determining causality is difficult in the absence of properly controlled comparisons. Loci harboring rare de novo and inherited copy number variants (CNVs), structural variations, and single nucleotide variants (SNVs) have all been described [5–7], comprising genes that functionally converge on synaptic function, chromatin re-modeling, and neuronal signaling and development [8, 9]. Although principally occurring sporadically or de novo, several pedigrees with inherited CNVs or point mutations in a number of key CNS genes or regions have also been reported, including *SHANK1*, *CDH8*, *NRXN3*, *PTCHD1*, and 16p11.2 [6]. In many of these, the parent transmitting the variant had related phenotypes, principally BAP. These case reports are potentially important, but each family on its own is insufficient to provide compelling evidence of association.

These ASD susceptibility loci represent the tip of the “heritable ASD” iceberg, with etiology remaining unknown for a substantial portion of ASD. Genetic modeling has suggested that at least 50% of the variance of the disorder may be due to common inherited variants [10], although the results of genome-wide association studies have been largely disappointing [11], with only one signal detected and replicated in a recent meta-analysis [12]. On the other hand, linkage studies using affected sib pairs have indicated many significant linkage peaks [6]; however, fine mapping has not been successful in uncovering genetic variants that clearly influence ASD risk under those linkage peaks. The current shift away from genome-wide linkage studies may be one reason the field has not made greater progress with respect to inherited variants. A comprehensive understanding of the genetic architecture of ASD requires unbiased knowledge about the number of causal loci, and at each locus the genetic models, effect sizes (i.e., penetrances), and allele frequencies of each identified variant. It is also important to know about interactions with other genes and between genes and environmental factors. To acquire this knowledge, studies are needed that focus on inherited variants and on a broad range of familial phenotypes including not only ASD but also BAP. Unfortunately, although some early genome-wide linkage studies of ASD and related traits were largely successful in the identification of signals [13–16], none have so far been followed up to examine underlying variants that segregate with phenotype. Although there has been a move away from the genome-wide linkage approach in ASD genetics, the known familial nature of ASD, coupled with the success of this approach in similar complex disorders [17, 18], lends support to methodological suitability to this disorder.

In the presence of high levels of locus heterogeneity (as is very likely in ASD), a potentially powerful approach to identify inherited loci is to study large families with many affected individuals who might share a single genetic locus of interest. Large families with several affected individuals are not uncommon in ASD, and a number of studies of such pedigrees have been published [14, 15, 19–22]. The attraction of using large extended pedigrees is based on the assumption that there is at least relative intrafamilial locus homogeneity if not locus homogeneity between families [23]. In these studies, potential regions of interest have been identified, but the linkage signals (with few exceptions, see Piven et al. [14]) have not been strong. Examining endophenotypes or more fundamental or broadly defined ASD-related traits, such as BAP, rather than diagnostic classification, is another potential way to increase power to detect variants of interest in studies of large pedigrees. Population-based twin studies have reported that there is no discontinuity between ASD and BAP in their genetic modeling [3] suggesting that ASD may simply be the extreme end of the distribution of autistic traits that constitute BAP. If that is the case, including individuals with BAP in a linkage study should increase power to identify loci [22].

In our previous study, we reported on 19 families, 6 recruited from Canada (CAN) and 13 recruited from the United States (US) [14, 19]. In this study, we focus on a set of 15 CAN pedigrees, including the 6 previously reported and 9 newly characterized pedigrees, and also consider results across all 28 (CAN and US) families. We consider both the ASD phenotype on its own as well as a phenotype that includes both ASD and BAP.

## Methods

### Participants

We recruited extended pedigrees with at least three ASD cases spread across at least two nuclear families. All families were either known to the authors through previous studies or identified through advertising. To minimize etiologic heterogeneity, families were excluded from the study if there was evidence of the following co-occurring medical conditions, thought to be etiologically related to autism, in one of the index probands: tuberous sclerosis, neurofibromatosis, phenylketonuria, fragile X syndrome, or significant CNS injury. We did not exclude individuals with a chromosome abnormality as detected by microarray in order to determine whether that abnormality might also be inherited and play a role in susceptibility; however, none were found. All individuals were of northern European ancestry. Data collection took place under Institutional Review Board approval, and the research was conducted in accordance with the World Medical Association Declaration of Helsinki. Written informed consent was obtained from subjects or their proxy decision-maker after the study had been fully explained.

### Clinical methods

Clinical assessments were performed to (1) index eligible extended pedigrees, by identifying at least three related individuals with a DSM-IV Pervasive Developmental Disorder, or DSM-5 ASD diagnosis across multiple nuclear families within a pedigree, and (2) characterize all relevant pedigree members on phenotypes of interest. For the latter goal, the strategy employed was to assess for both ASD or, in non-ASD individuals, BAP. The goal in taking this multi-tiered approach was to maximize the aggregate information available on the maximum number of affected individuals (i.e., global ratings of ASD or BAP).

Overlapping sets of instruments were used to diagnose ASD and BAP in the pedigrees. After initial telephone screening, the Autism Family History Interview (AFHI) [24] was administered and a review of medical records was conducted to confirm a presumptive diagnosis of ASD. This diagnosis was subsequently confirmed by expert clinical judgment incorporating information from the Autism Diagnostic Interview Revised (ADI-R) [25] and Autism Diagnostic Observation Schedule-Revised (ADOS-R) [26], which were administered by trained and reliable clinicians. All participants classified as ASD met DSM IV criteria for either Autistic Disorder, Asperger syndrome, or Pervasive Developmental Disorder Not Otherwise Specified (PDDNOS) according to the criteria in Risi et al. [27]. Non-ASD family members were prioritized so that information was obtained from first-degree relatives of ASD individuals or relatives in the blood line between two such individuals.

In the CAN pedigrees, the Broader Autism Phenotype Questionnaire (BAP-Q) [28] was used for diagnosis of BAP in individuals greater than 15 years of age. The measure was completed by the participant about him/herself (the self-version) and also by someone close to the participant about him/her (parent or spouse, the informant version) to obtain an average score (between the self and informant scores). Whenever available, the average scores were utilized. A BAP diagnosis was assigned if an individual met gender-specific criteria in any domain. Higher diagnostic cutoffs with higher specificity than those used in clinical practice were used in screening [29].

Consensus ratings based on The Modified Personality Assessment Schedule Revised (MPAS-R) and Modified Pragmatic Rating Scale (MPRS) were used to identify BAP in the US pedigrees as described previously [30, 31]. When MPAS-R and MPRS consensus ratings were not available, BAPQ was used for BAP diagnosis.

### Genotyping and data cleaning

#### Canadian pedigrees

Three hundred thirty-four individuals from the Canadian data set were genotyped using either the Illumina Omni 2.5M chip (6 families) or the Illumina HumanCoreExome chip (9 families). SNP data were used to verify family structure, and founders were assessed for relatedness (no relatedness was found). Thirteen individuals were dropped due to unresolvable relationship issues. Genotypes were cleaned for missingness by marker and by individual, dropping 2823 markers with > 5% missing data, but no individuals had > 5% missing data. Data were checked for Mendel errors, again using a threshold of 5%. Two additional markers were dropped due to excess Mendel errors, but no individuals were dropped. Remaining Mendel errors were removed by changing genotypes within the family to missing for the SNP in question. Twenty-four SNPs with a Hardy-Weinberg (HW) *p* value < 1 × 10^−4^ were also dropped. All remaining markers were used to call CNVs. CNVs > 15 kb in length, supported by five or more probes and identified by two or more algorithms, were considered. CNVs were further filtered to identify only those that were rare in the population; none were observed to be segregating under linkage peaks. After data cleaning, a total of 529 individuals remained (mean per pedigree = 35.3, s.d. = 13.9, minimum = 15, maximum = 56), of whom 321 were genotyped (mean per pedigree = 21.4, s.d. = 8.9; minimum = 10, maximum = 37) and 234 were phenotyped (mean per pedigree = 15.6, s.d. = 4.7; minimum = 9, maximum = 24).

In preparation for linkage analysis, a marker selection protocol [32] was applied to a baseline set of 210,716 common markers (present on both chips) in order to thin the map to remove marker-to-marker LD (*r*^2^ > 0.20). This resulted in a reduced set of 22,004 SNPs (minor allele frequency mean = 0.45, s.d. = 0.06; intermarker distance mean = 0.17 cM, s.d. = 0.14). The Rutgers Combined Linkage-Physical Map (http://compgen.rutgers.edu/) (custom release May 2014; Build 37 hg19) [33] was used to place the SNPs on a genetic (cM) map.

#### US pedigrees

Thirteen US families as described in [14, 19] were previously analyzed. Briefly, these comprised 309 individuals (mean = 23.8 per pedigree, s.d. = 11.5), of whom 187 were genotyped (mean = 14.4 per pedigree, s.d. = 7.5) using a dense microsatellite marker set combined with SNP data from the Illumina Omni 2.5M chip; similar data preparation protocols were used. Here, we take advantage of the PPL to sequentially update genome-wide linkage results between the current set of CA pedigrees and the previously analyzed US pedigrees.

### Statistical methods

Linkage analysis was conducted using the software package KELVIN (v2.4.9), which implements the PPL (posterior probability of linkage) class of models for measuring the strength of genetic evidence [34]. In order to take advantage of the very dense marker coverage in a multipoint setting, and given the size of the pedigrees, MCMC was used to calculate marker likelihoods as described in [35], while KELVIN’s non-stochastic algorithm was used to calculate trait likelihoods conditional on marker data [36].

Two different dichotomous traits were employed: ASD and ASD with BAP. Our decision to analyze dichotomous rather than continuous traits was due to the current lack of a psychometrically validated measure that characterizes the range of ASD and BAP symptoms and their relationship to categorical diagnoses among diagnosed and non-diagnosed individuals. When analyzing ASD, BAP individuals were coded as unaffected; when analyzing BAP, both ASD and BAP individuals were considered affected. The model is parameterized in terms of *α* (the admixture parameter of Smith [37], representing the proportion of “linked” pedigrees), *p* (the disease allele frequency), and the penetrance vector *f*_*i*_, representing the probability that an individual with genotype *i* develops disease, for *i* – 1..3. All trait parameters are integrated out of the final statistic, using essentially uniform prior distributions (ordering constraints are imposed on the penetrances [34, 38]), implicitly allowing for dominant, recessive, and additive models. This provides a robust approximation for mapping complex traits in terms of the marginal model at each locus, and because the parameters are integrated out, no specific assumptions regarding their values are required. The method implicity allows for phenocopies.

The PPL has two basic approaches to the accumulation of evidence, which we employ here to consider evidence across pedigrees. Under “pooled” (PPL_POOL_), the trait parameters are integrated over across all pedigrees as a set at each locus. This is appropriate under the expectation that at each locus, the trait model is essentially the same across pedigrees. Under “sequential” (PPL_SEQ_), the trait parameters are integrated over separately for each pedigree at each locus, and the marginal evidence for or against linkage itself is accumulated across pedigrees using Bayesian sequential updating. Sequential updating is appropriate under the expectation that each pedigree may implicate different loci and or the same loci but under different trait models (as could arise, e.g., in the presence of important background genetic and/or environmental modification). When there is relative genetic homogeneity, pooling will yield larger signals at linked loci; when there is extensive heterogeneity, however, sequential updating will yield larger signals at linked loci and also smaller signals at unlinked ones [39]. Sequential updating can also be used to accumulate evidence for or against linkage across multiple *sets* of families, as we do below in combining results from the CA pedigrees with the previously analyzed US pedigrees.

The PPL is on the probability scale, and its interpretation is therefore fairly straightforward, e.g., PPL = 40% means that there is an estimated 40% probability of a trait gene at the given location based on the data. The only caveat to this interpretation is that this estimated probability is influenced by the low prior probability of linkage (*π*) to any given locus. Based on empirical data [40], we set *π* = 2%. (This assumes just one disease gene in the genome and is thus conservative, possibly highly conservative, under locus heterogeneity.) Thus, PPL > 2% indicates some degree of evidence in favor of a trait gene at that locus, while PPL < 2% represents evidence against the location. As with any Bayesian method, the influence of this small prior probability on the final PPL can be appreciable until the data set becomes large. For this reason, it is helpful to interpret the PPL by comparison with the prior: a PPL of, say, 20% indicates that the data are supporting linkage enough to make the posterior probability 10 times larger than the prior probability of 2%.

Additional distinctive features of the statistical framework are related to the fact that the PPL is a measure of statistical evidence, not a decision-making procedure. There are, therefore, no “significance levels” associated with it (i.e., no specific cutoffs beyond which we declare significance), and it is not interpreted in terms of associated error probabilities [41]. By the same token, no multiple testing corrections are applied to the PPL, just as one would not “correct” a measure of the temperature made in one location for temperature readings taken at different locations [42]. The reader may be assisted in their interpretation of the results by recognizing that the Bayes ratio (BR) used in PPL calculations, i.e., the ratio of probabilities for the null versus alternative hypotheses, is very closely related to the exponentiated LOD. However, in calculating the BR, the trait parameters are not fixed at particular values but, rather, are integrated out of the underlying likelihood ratio prior to transformation via Bayes theorem onto the posterior probability (PPL) scale.

## Results

Fifteen CA pedigrees met the inclusion criteria (Additional file 1: Table S1). In all but one pedigree, the three cases were spread across three nuclear families. There was an average of five ASD individuals per pedigree (s.d. = 1.7, range 3–8), four BAP individuals per pedigree (s.d. = 3.32, range 1–12), and eight ASD + BAP individuals per pedigree (s.d. = 3.85, range 4–15). The prevalence of BAP among all phenotyped individuals is therefore 22.6%; including all ASD cases in the definition of BAP raises this to 53.8%. Consistent with the collection of BAPQ data on individuals aged 16 and over, the ASD cases were younger than those identified as BAP (ASD mean age 11.7 years, range 1.8–56 years; BAP mean age 43.6 years, range 15.4–84.3 years), with a male to female ratio of ~ 6:1 and 1:1.4 for ASD and BAP cases respectively (more detailed characteristics of the sample, including IQ and adaptive function, are available in Additional file 1: Table S1). The 13 US pedigrees comprised an average of four ASD individuals per pedigree (s.d. = 1.14, minimum = 3, maximum = 6) and an average of five BAP individuals per pedigree (s.d. = 2.58, minimum = 2, maximum = 10) as previously described [14].

We first examined the CA pedigrees separately. Consistent with our previous report [14], we again found that for both ASD and “ASD + BAP”, sequential updating provided stronger signals than pooling, consistent with considerable locus heterogeneity between pedigrees (Additional file 1: Figure S1). As in Piven et al. [14], we therefore focus on sequentially updated results in the remainder of the paper. Additionally, sequentially updated linkage signals provide evidence of larger peaks and more numerous signals clearly visually separable from background noise for “ASD + BAP” (Additional file 1: Figure S2). Given the likelihood of substantial interfamilial heterogeneity, results in each individual pedigree considered on its own are of interest (Additional file 1: Figures S3 and S4). Overall, with the exception of Ped 6 (see Additional file 1: Figure S4), individual pedigrees do not show compelling evidence of linkage, similar to what was reported in Piven et al. [14]. Given the size of the pedigrees, this is consistent with heterogeneity between pedigrees as well as moderate to high levels of heterogeneity *within* the pedigrees. Within-pedigree heterogeneity may be due to multiple disease loci segregating within the same pedigree or the presence of (non-genetic) phenocopies. However, the relative contributions of these are very difficult to estimate reliably.

**Fig. 1:**
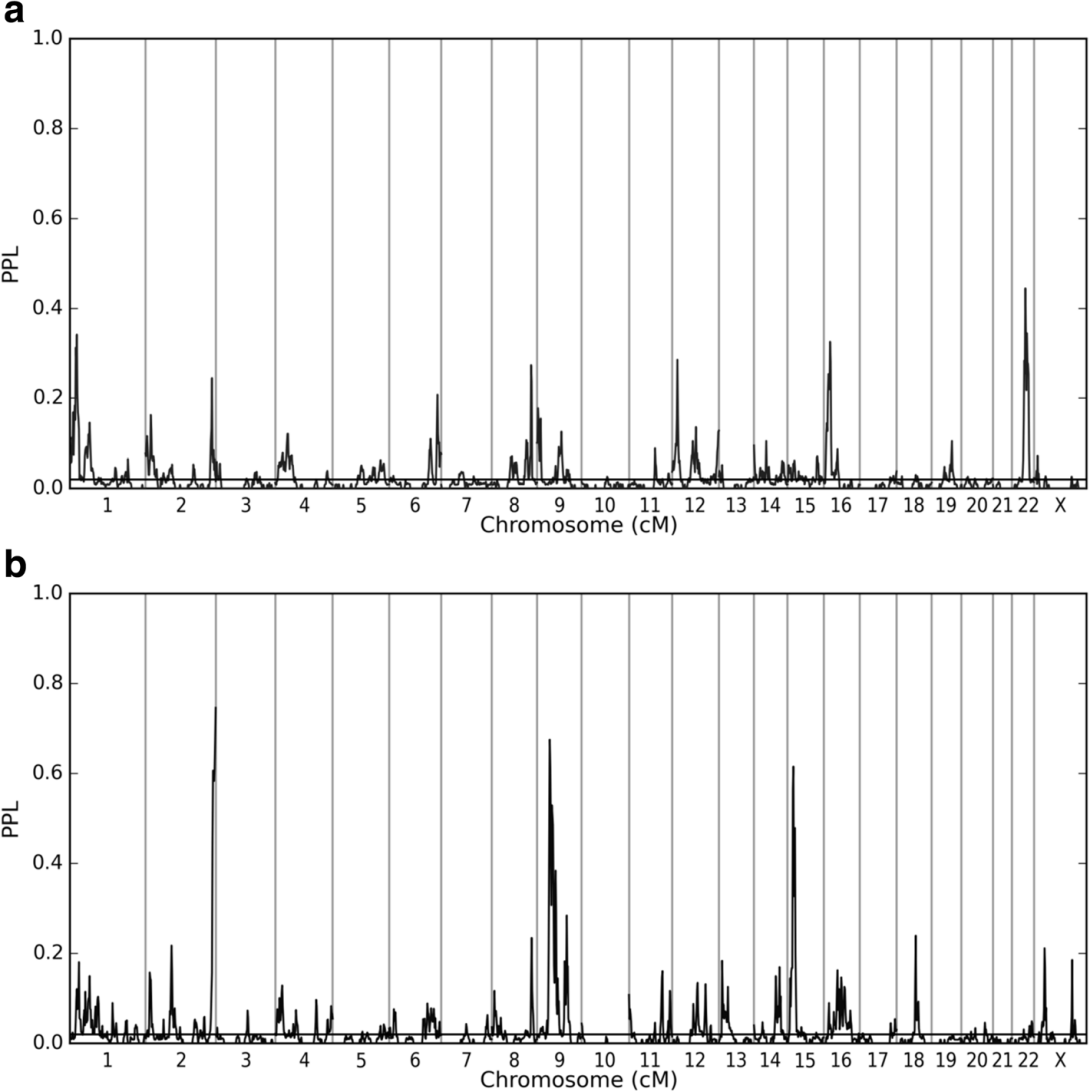
Sequentially updated combined CAN and US pedigrees. **a** ASD and **b** BAP results sequentially updated for combined CAN and US pedigrees. Note that the *y*-axis goes from 0.0–1.0

We next combined these 15 CAN pedigrees with 13 US pedigrees. Using all 28 pedigrees across the two studies, we obtain multiple peaks that clearly stand out from background noise (Fig. 1), for both ASD and “ASD + BAP.” Genome-wide ASD and “ASD + BAP” results are somewhat correlated, with “ASD + BAP” returning the highest scores. There are a number of “ASD + BAP” peaks that drop when the data are analyzed under ASD phenotype, as would be expected when recoding “affecteds” (i.e., BAP cases) as unaffected. However, in contrast to what would be expected under a common ASD/BAP locus model, several loci emerge for which the inclusion of BAP cases *reduces* the linkage signal, including two for which including BAP cases yield evidence against linkage, suggesting that these loci may be ASD specific.

The occurrence of some substantially larger signals when we sequentially update across both the CAN and US pedigrees is consistent with shared loci across families even given appreciable intrafamilial heterogeneity. Table 1 gives details (Additional file 1: Table S2 provides genes in these linked regions that have been previously implicated in ASD).

**Table 1:** Salient ASD, BAP linkage peaks, and CAN and US pedigrees

Chr & Band	ASD PPL (%)	BAP PPL (%)	Peak (cM)^a^	Narrow^b^	Intermed	Broad	Peak (BP position)^c^
1p36.22	*34*	12	26	22–28	16–34	0–34	11,957,977
2q37.2	*25*	16	250	250	246–250	244–256	236,361,323
6q27	*21*	2	182	182	180–184	178–188	165,645,201
8q24.22	*27*	14^4^	148	148–150	148–150	144–150	134,467,348
12p13.31	*29*	1.5	20	20	18–22	10–28	7,531,425
16p13.2	*33*	4	24	16–26	10–28	8–28	9,330,226
22q13.1	*45*	1.5	50	46–62	44–62	42–64	37,698,639
2p13.1	3	*22*	98	98	96–100	92–104	74,913,089
2q37.3	2	*75*	264	252–264	248–264	246–264	243,361,159
9p21.3	1.6	*67*	48	44–62	44–72	42–82	24,428,328
9q31.2	4	*28*	112	112	110–116	102–118	109,889,954
15q13.3	6	*62*	22	20–28	14–32	10–32	31,770,967
18q21.1	3	*24*	72	72	70–72	70–82	45,574,928
Xp22.11	2	*21*	40	40	40–42	38–46	22,295,443

^a^Both ASD and BAP PPLs are shown at the same location, corresponding to the location for the phenotype with the larger PPL (indicated in italics)

^b^Peak width in cM, defined as the contiguous region around the peak for which the PPL remains ≥ 0.20 (narrow), ≥ 0.10 (intermediate), or ≥ 0.05 (broad), for the phenotype with the larger PPL

^c^Physical positions reference Build 37 and are included for convenience only; linkage analysis has a resolution of approximately 1 cM (on average, around 1 M basepairs) at best

**Fig. 2:**
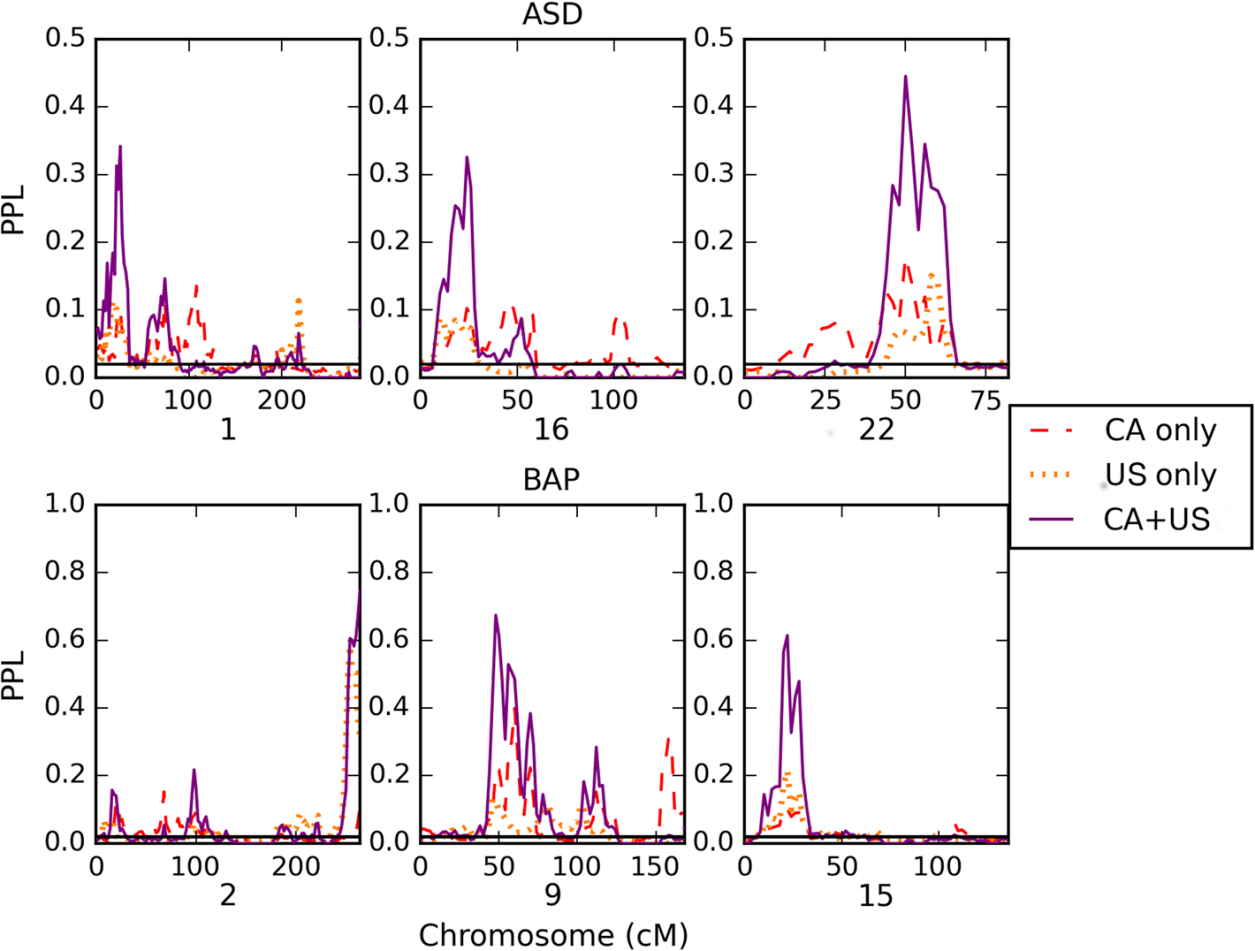
Accumulation of evidence across CAN and US pedigrees. Accumulation of evidence across CAN and US pedigrees. Shown here are all chromosomes with CAN + US sequentially updated PPL ≥ 0.30. For visual clarity, the *y*-axis goes from 0 to 0.5 for ASD and 0–1.0 for BAP

Figure 2 shows details of the most salient linkage peaks.

As Fig. 2 shows, localization of linkage peaks is imprecise and variable between CAN and US pedigrees across pedigrees. Despite this, however, when we consider all 28 families together, we do begin to see some notable linkage signals (Fig. 2). Note too that by design, the PPL becomes larger at linked loci as more data are available, and also, it becomes smaller at unlinked loci, in a model-free manner as noted above. For CAN alone, 57% (when ASD is the phenotype) of the genome and 69% (when “BAP + ASD” is used) give evidence against linkage (PPL ≤ 0.02), while for CAN + US, the corresponding numbers are 71 and 72% for ASD and “ASD + BAP” respectively.

## Discussion

The objective of this paper was to identify chromosomal regions containing inherited genetic variants for ASD. We employed three methods to accomplish this objective: (1) we ascertained large, extended pedigrees with at least three individuals affected with ASD; (2) we assessed for BAP to identify a greater number of affected subjects within a pedigree; and (3) we employed statistical methods tailored to this application, especially insofar as they can distinguish evidence for linkage from evidence against linkage in the face of genetic heterogeneity. Our expectations were that, compared to linkage studies using affected sib pairs, each of these pedigrees would be enriched for one (or a small number of) segregating variants or, in other words, that there would be greater homogeneity within if not between pedigrees and that the inclusion of BAP would allow for greater power to identify linked genomic regions.

We were able to replicate our earlier finding that sequential updating of linkage evidence was more informative than pooling all pedigrees [14], implying that there remains considerable between-pedigree heterogeneity. We again found that few if any of the individual pedigrees on their own provided compelling evidence of linkage, suggesting the presence of considerable within-pedigree heterogeneity as well [43]. It is particularly interesting, then, that despite what are likely to be high levels of both inter- and intra-pedigree heterogeneity, sequentially updating with the US pedigrees led to several salient linkage peaks both for ASD and “ASD + BAP” phenotypes. This suggests that while multiple susceptibility variants segregate in these pedigrees, the number of variants is not so high as to preclude overlapping loci (not necessarily variants) across pedigrees. Notably, these results also suggest loci for which ASD and BAP share genetic etiology, as well as loci that may be ASD specific.

A number of the loci identified by the combined CAN and US families overlap genes implicated in ASD, as well as DECIPHER syndromes identified as “grade 1,” thereby earmarking them for their strong evidence of pathogenicity [44]. In particular, the locus at 1p36.22 (ASD PPL = 34%) overlaps the 1p36 microdeletion syndrome implicated in a severe syndrome of intellectual disability, epilepsy, and craniofacial dysmorphology, with other body systems variably impacted [45]. Additionally, both ASD (PPL = 25%) and “ASD + BAP” (PPL = 75%)-specific signals overlap the 2q37 microdeletion locus, which is also characterized by syndromal intellectual disability [46]. The Xp22.11 chromosome signal overlaps *PTCHD1* [47]. Finally, a further signal overlaps the 16p13.11 neurocognitive disorder susceptibility locus [48]. As is evident in Additional file 1: Table S2, these and other loci we have identified overlap a number of genes implicated in ASD and as such are targets for further investigation for a possible role for underlying variants in heritable ASD. Note that we did not observe segregating CNVs under these peaks. An examination of CNVs and single nucleotide variants within these families will be the subject of a further paper.

It is interesting to compare the results of this study with those using an identical PPL method in the analysis of a data set incorporating 1129 trios and 1069 multiplex families (the Autism Genome Project [AGP] [49]). The first observation is that none of the signals between these two independent samples overlap, although acknowledging the likely interfamilial heterogeneity, this is not unsurprising. What is most striking is that despite the much larger sample size in the Vieland et al. study, the signals are similar in size to those identified in our analysis. It has previously been demonstrated that, in reference to variance component linkage methods, extended pedigrees offer superior power compared to smaller nuclear families, irrespective of the underlying genetic architecture [50]. Comparing the results of our extended pedigrees with the AGP data is therefore consistent with this finding and supports the role for such pedigrees in the future search for heritable causes of ASD.

In spite of the strengths of the study, there are several limitations that should be taken into account. Multiple disease alleles might be brought in by married-ins within each pedigree, due to ascertainment and/or assortative mating [51]. This phenomenon would explain the finding of small peaks within each pedigree even given the large number of affected subjects. We are not aware, however, of evidence of assortative mating in ASD or BAP [52]. On the other hand, despite the small signals per pedigree, sequentially updating over all 28 pedigrees yields multiple notable linkage peaks, suggesting that collection of additional multiplex pedigrees to improve power—together with phenotyping of non-ASD relatives—remains an attractive strategy for resolving the genetics of inherited forms of ASD. Indeed, together with the inclusion of subclinical phenotypes and/or endophenotypes such as BAP, a study design such as ours offers a powerful approach to provide genetic loci that can then be targeted by high-coverage sequencing. Arguably therefore, the identification of heritable genetic causes of ASD will benefit from an approach that once again incorporates techniques such as genome-wide linkage into a larger methodological framework. In particular, linkage analysis can be an effective filtering strategy for whole-genome sequencing studies by allowing regions with evidence of linkage to form the focus of a more comprehensive analysis. Alternatively, in a genome-wide rare variant association framework, variants in a linked region could be given greater weight. Either way, incorporating evidence from linkage presents a useful strategy to improve the power to detect heritable rare variants in ASD genetics [53]. The next step, therefore, is to follow up on the identified regions in these pedigrees by way of whole-genome sequencing and analyzing the data in the ways suggested above. This may also help to resolve the cause of heterogeneity both between and within pedigrees.

## Conclusions

Extended pedigrees offer superior power compared to smaller nuclear families in the identification of loci harboring heritable ASD and BAP variants, with both ASD and combined ASD/BAP-specific loci apparent. However, the results also confirm appreciable interfamilial heterogeneity as well as a high level of intrafamilial heterogeneity. Inclusion of subclinical phenotypes such as BAP should be more widely employed in genetic studies of ASD as a way of identifying inherited genetic variants for the disorder. Moreover, the results underscore the need for approaches to identifying genetic risk factors in extended pedigrees that are robust to high levels of inter/intrafamilial locus and allelic heterogeneity.

## Additional file

Additional file 1: Phenotypes and additional genetic plots. (PDF 2638 kb)

## Abbreviations

AGP: Autism Genome Project
ASD: Autism spectrum disorder
BAP: Broad autism phenotype
CNV: Copy number variant
ID: Intellectual disability
PPL: Posterior probability of linkage

## References

[1] Ozonoff S, Young GS, Carter A, Messinger D, Yirmiya N, Zwaigenbaum L (2011). Recurrence risk for autism spectrum disorders: a baby siblings research consortium study. Pediatrics.

[2] Centers for Disease Control and Prevention (2012). Prevalence of autism spectrum disorders—autism and developmental disabilities monitoring network, 14 Sites, United States, 2008. Surveil Summ.

[3] Tick B, Bolton P, Happe F, Rutter M, Rijsdijk F (2016). Heritability of autism spectrum disorders: a meta-analysis of twin studies. J Child Psychol Psychiatry.

[4] Sasson NJ, Lam KS, Parlier M, Daniels JL, Piven J (2013). Autism and the broad autism phenotype: familial patterns and intergenerational transmission. J Neurodev Disord.

[5] Devlin B, Scherer SW (2012). Genetic architecture in autism spectrum disorder. Curr Opin Genet Dev.

[6] Geschwind DH, State MW (2015). Gene hunting in autism spectrum disorder: on the path to precision medicine. Lancet Neurol.

[7] Bourgeron T (2015). From the genetic architecture to synaptic plasticity in autism spectrum disorder. Nat Rev Neurosci.

[8] Pinto D, Delaby E, Merico D, Barbosa M, Merikangas A, Klei L (2014). Convergence of genes and cellular pathways dysregulated in autism spectrum disorders. Am J Hum Genet.

[9] RK CY, Merico D, Bookman M, LH J, Thiruvahindrapuram B, Patel RV (2017). Whole genome sequencing resource identifies 18 new candidate genes for autism spectrum disorder. Nat Neurosci.

[10] Gaugler T, Klei L, Sanders SJ, Bodea CA, Goldberg AP, Lee AB (2014). Most genetic risk for autism resides with common variation. Nat Genet.

[11] Warrier V, Chee V, Smith P, Chakrabarti B, Baron-Cohen S (2015). A comprehensive meta-analysis of common genetic variants in autism spectrum conditions. Mol Autism..

[12] Autism Spectrum Disorders Working Group of The Psychiatric Genomics C (2017). Meta-analysis of GWAS of over 16,000 individuals with autism spectrum disorder highlights a novel locus at 10q24.32 and a significant overlap with schizophrenia. Mol Autism..

[13] Szatmari P (2007). Mapping autism risk loci using genetic linkage and chromosomal rearrangements. Nat Genet.

[14] Piven J, Vieland VJ, Parlier M, Thompson A, O'Conner I, Woodbury-Smith M (2013). A molecular genetic study of autism and related phenotypes in extended pedigrees. J Neurodev Disord.

[15] Coon H, Villalobos ME, Robison RJ, Camp NJ, Cannon DS, Allen-Brady K (2010). Genome-wide linkage using the social responsiveness scale in Utah autism pedigrees. Mol Autism.

[16] Liu XQ, Paterson AD, Szatmari P (2008). Genome-wide linkage analyses of quantitative and categorical autism subphenotypes. Biol Psychiatry.

[17] Bowden DW, An SS, Palmer ND, Brown WM, Norris JM, Haffner SM (2010). Molecular basis of a linkage peak: exome sequencing and family-based analysis identify a rare genetic variant in the ADIPOQ gene in the IRAS family study. Hum Mol Genet.

[18] Rosenthal EA, Ronald J, Rothstein J, Rajagopalan R, Ranchalis J, Wolfbauer G (2011). Linkage and association of phospholipid transfer protein activity to LASS4. J Lipid Res.

[19] Woodbury-Smith M, Paterson AD, Thiruvahindrapduram B, Lionel AC, Marshall CR, Merico D (2015). Using extended pedigrees to identify novel autism spectrum disorder (ASD) candidate genes. Hum Genet.

[20] Allen-Brady K, Robison R, Cannon D, Varvil T, Villalobos M, Pingree C (2010). Genome-wide linkage in Utah autism pedigrees. Mol Psychiatry.

[21] Woodbury-Smith M, Bilder DA, Morgan J, Jerominski L, Darlington T, Dyer T (2017). Combined genome-wide linkage and targeted association analysis of head circumference in autism spectrum disorder families. J Neurodev Disord.

[22] Chapman NH, Nato AQ, Bernier R, Ankenman K, Sohi H, Munson J (2015). Whole exome sequencing in extended families with autism spectrum disorder implicates four candidate genes. Hum Genet.

[23] Wijsman EM (2012). The role of large pedigrees in an era of high-throughput sequencing. Hum Genet.

[24] Piven J, Gayle J, Chase GA, Fink B, Landa R, Wzorek MM (1990). A family history study of neuropsychiatric disorders in the adult siblings of autistic individuals. J Am Acad Child Adolesc Psychiatry.

[25] Lord C, Rutter M, Le Couteur A (1994). Autism Diagnostic Interview-Revised: a revised version of a diagnostic interview for caregivers of individuals with possible pervasive developmental disorders. J Autism Dev Disord.

[26] Lord C, Risi S, Lambrecht L, Cook EH, Leventhal BL, DiLavore PC (2000). The autism diagnostic observation schedule-generic: a standard measure of social and communication deficits associated with the spectrum of autism. J Autism Dev Disord.

[27] Risi S, Lord C, Gotham K, Corsello C, Chrysler C, Szatmari P (2006). Combining information from multiple sources in the diagnosis of autism spectrum disorders. J Am Acad Child Adolesc Psychiatry.

[28] Hurley RS, Losh M, Parlier M, Reznick JS, Piven J (2007). The broad autism phenotype questionnaire. J Autism Dev Disord.

[29] Sasson NJ, Lam KS, Childress D, Parlier M, Daniels JL, Piven J (2013). The broad autism phenotype questionnaire: prevalence and diagnostic classification. Autism Res.

[30] Losh M, Adolphs R, Poe MD, Couture S, Penn D, Baranek GT (2009). Neuropsychological profile of autism and the broad autism phenotype. Arch Gen Psychiatry.

[31] Losh M, Piven J (2007). Social-cognition and the broad autism phenotype: identifying genetically meaningful phenotypes. J Child Psychol Psychiatry.

[32] Vieland VJ, Walters KA, Azaro M, Brzustowicz LM, Lehner T (2014). The value of regenotyping older linkage data sets with denser marker panels. Hum Hered.

[33] Matise TC, Chen F, Chen W, De La Vega FM, Hansen M, He C (2007). A second-generation combined linkage physical map of the human genome. Genome Res.

[34] Vieland VJ, Huang Y, Seok SC, Burian J, Catalyurek U, O'Connell J (2011). KELVIN: a software package for rigorous measurement of statistical evidence in human genetics. Hum Hered.

[35] Huang Y, Thomas A, Vieland VJ (2013). Employing MCMC under the PPL framework to analyze sequence data in large pedigrees. Front Genet.

[36] Seok SC, Evans M, Vieland VJ (2009). Fast and accurate calculation of a computationally intensive statistic for mapping disease genes. J Comput Biol.

[37] Smith CA (1963). Testing for heterogeneity of recombination fraction values in human genetics. Ann Hum Genet.

[38] Bartlett CW, Vieland VJ (2007). Accumulating quantitative trait linkage evidence across multiple datasets using the posterior probability of linkage. Genet Epidemiol.

[39] Govil M, Vieland VJ (2008). Practical considerations for dividing data into subsets prior to PPL analysis. Hum Hered.

[40] Elston RC, Lange K (1975). The prior probability of autosomal linkage. Ann Hum Genet.

[41] Vieland VJ (1998). Bayesian linkage analysis, or: how I learned to stop worrying and love the posterior probability of linkage. Am J Hum Genet.

[42] Vieland VJ (2006). Thermometers: something for statistical geneticists to think about. Hum Hered.

[43] Yuen RK, Thiruvahindrapuram B, Merico D, Walker S, Tammimies K, Hoang N (2015). Whole-genome sequencing of quartet families with autism spectrum disorder. Nat Med.

[44] Firth HV, Richards SM, Bevan AP, Clayton S, Corpas M, Rajan D (2009). DECIPHER: database of chromosomal imbalance and phenotype in humans using Ensembl resources. Am J Hum Genet.

[45] Battaglia A, Hoyme HE, Dallapiccola B, Zackai E, Hudgins L, McDonald-McGinn D (2008). Further delineation of deletion 1p36 syndrome in 60 patients: a recognizable phenotype and common cause of developmental delay and mental retardation. Pediatrics.

[46] Falk RE, Casas KA (2007). Chromosome 2q37 deletion: clinical and molecular aspects. Am J Med Genet C Semin Med Genet.

[47] Noor A, Whibley A, Marshall CR, Gianakopoulos PJ, Piton A, Carson AR (2010). Disruption at the PTCHD1 locus on Xp22.11 in autism spectrum disorder and intellectual disability. Sci Transl Med.

[48] Ramalingam A, Zhou XG, Fiedler SD, Brawner SJ, Joyce JM, Liu HY (2011). 16p13.11 duplication is a risk factor for a wide spectrum of neuropsychiatric disorders. J Hum Genet.

[49] Vieland VJ, Hallmayer J, Huang Y, Pagnamenta AT, Pinto D, Khan H (2011). Novel method for combined linkage and genome-wide association analysis finds evidence of distinct genetic architecture for two subtypes of autism. J Neurodev Disord.

[50] Williams JT, Van Eerdewegh P, Almasy L, Blangero J (1999). Joint multipoint linkage analysis of multivariate qualitative and quantitative traits. I. Likelihood formulation and simulation results. Am J Hum Genet.

[51] Durner M, Greenberg DA, Hodge SE (1992). Inter- and intrafamilial heterogeneity: effective sampling strategies and comparison of analysis methods. Am J Hum Genet.

[52] Robinson MR, Kleinman A, Graff M, Vinkhuyzen AAE, Couper D, Miller MB (2017). Genetic evidence of assortative mating in humans. Nat Hum Behav.

[53] Yoo YJ, Bull SB, Paterson AD, Waggott D, Sun L, Diabetes C (2010). Were genome-wide linkage studies a waste of time? Exploiting candidate regions within genome-wide association studies. Genet Epidemiol.

