# Supplementary material for: A genome-wide linkage study of autism spectrum disorder and the broad autism phenotype in extended pedigrees
Source: J Neurodev Disord. 2018 Jun 11;10:20. doi: 10.1186/s11689-018-9238-9 (PMC5996536; doi:10.1186/s11689-018-9238-9)
Supplement: Supplementary file 1 — Phenotypes and additional genetic plots. (PDF 2638 kb) [file 11689_2018_9238_MOESM1_ESM.pdf]

A genome-wide linkage study of autism spectrum disorder and the broad autism phenotype in extended pedigrees

Short title: Genome-wide linkage in autism extended pedigrees

Marc Woodbury-Smith, Andrew D. Paterson, Irene O'Conner, Mehdi Zarrei, Ryan K. C. Yuen, Jennifer Howe, Ann Thompson, Morgan Parlier, Bridget Fernandez, Joseph Piven, Stephen W. Scherer, Veronica Vieland and Peter Szatmari

Table S1: Phenotypes for ASD and BAP cases for CAN pedigrees

	**ASD (N=73)**	**BAP (N=53)**
Sex: M:F	6.3:1	1:1.4
Age (months)[1] mean, SD	140.39, 120.73	523.35, 188.65
IQ (combined across measures) [2] N	61	4
Age (months) mean, SD	135.34, 88.77	348.79, 144.76
Verbal, N	40	3
Standard score mean, SD	96.01, 21.16	70.33, 5.69
Non-verbal, N	60	4
Standard score mean, SD	97.33, 21.12	88.50, 22.22
Full scale, N	41	3
Standard score mean, SD	95.73, 20.82	74.67, 15.04
Vineland Adaptive Behavior Scale, N	57	3[2]
Age (months) mean, SD	123.09, 80.18	376.10, 163.65
Communication: Standard score mean, SD	78.91, 19.28	65.67, 20.21
Daily living: Standard score mean, SD	74.49, 20.43	81.33, 30.19
Social: Standard score mean, SD	70.58, 16.09	79.33, 17.90
Adaptive behavior composite: Standard score mean, SD	72.67, 17.29	74.33, 24.00
BAP-Q Informant, N	6	44
Age (months) mean, SD	307.23, 133.66	516.45, 196.35
Aloof mean, SD	4.29, 1.09	3.09, 1.09
Pragmatic language mean, SD	3.47, 1.01	2.53, 0.96
Rigid mean, SD	4.40, 0.89	3.55, 1.02

[1]Age represents the age on completion of first assessments.

[2]Different IQ measures were used (Wechsler, Leiter, Stanford-Binet, Mullen), and combined to generate these summary scores. The verbal score was generated from either a verbal standard score or a verbal comprehension composite score (VCI) from a Wechsler, either the verbal standard score or the verbal reasoning SAS from a Stanford-Binet, or an expressive language t-score converted to a standard score from a Mullen. Similarly, nonverbal scores were generated from either a performance standard score or a perceptual reasoning composite score (PRI) from a Wechsler, the Leiter-R standard score, either the non-verbal standard score or the abstract/visual reasoning SAS from a Stanford-Binet, or a receptive language t-score converted to a standard score from a Mullen. Finally, the following indices were combined to generate full scale scores: a full scale standard score from a Wechsler, either a composite standard score or an abbreviated standard score from a Stanford-Binet, or an early learning composite score from a Mullen

Supplementary Figure 1 Pooling versus Sequential Updating for ASD in Canadian Pedigrees (a) Sequentially updated PPLs, (b) Pooled PPLs. The horizontal line at PPL = 0.02 indicates the dividing point between evidence for linkage (PPL > 0.02) and evidence against linkage (PPL < 0.02). Note that for visual clarity, the y-axis goes from 0.0-0.5, rather than from 0.0-1.0.

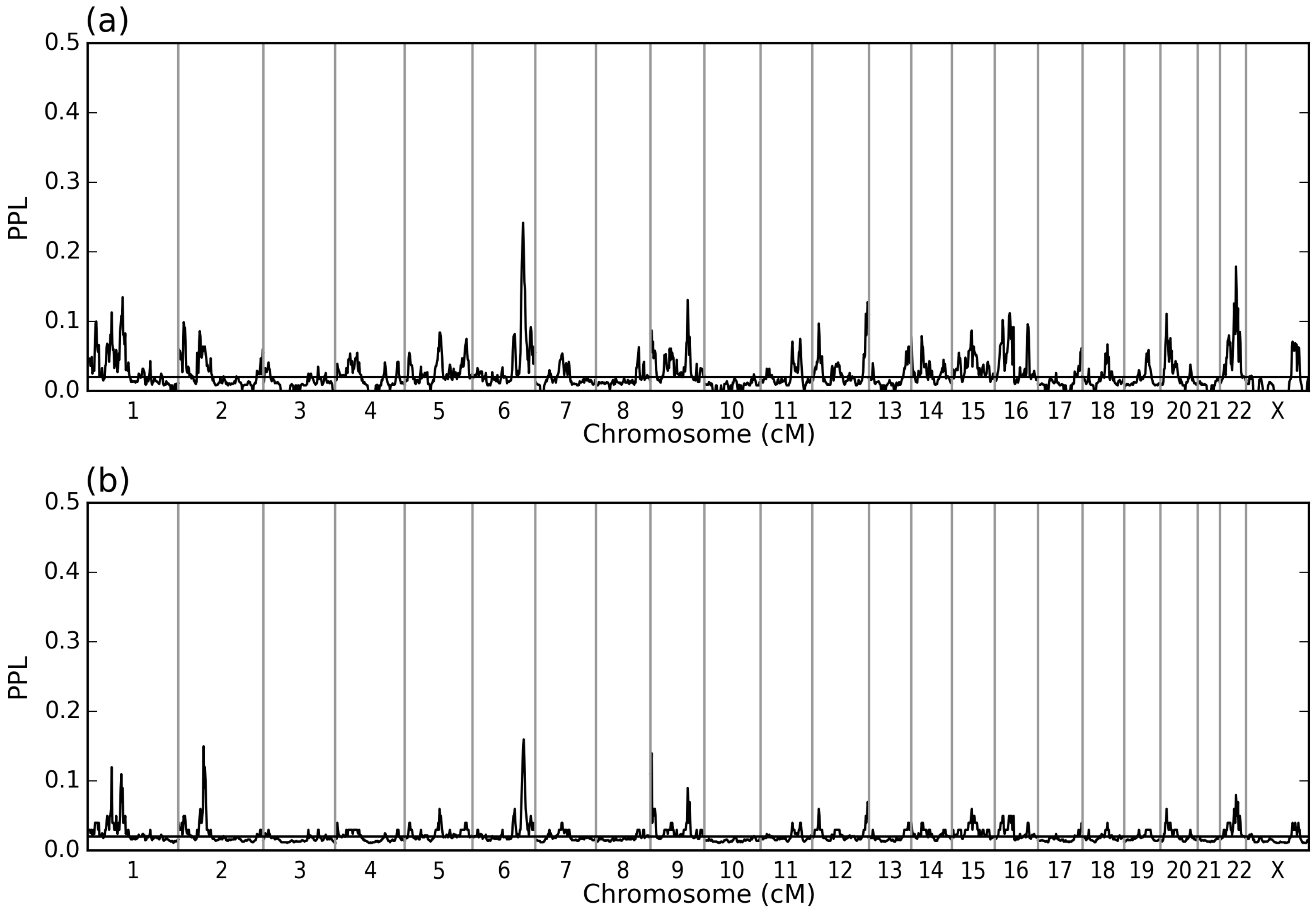

Supplementary Figure 2 Pooling versus Sequential Updating for BAP in Canadian Pedigrees (a) Sequentially updated PPLs, (b) Pooled PPLs. Note that for visual clarity, the y-axis goes from 0.0-0.5, rather than from 0.0-1.0

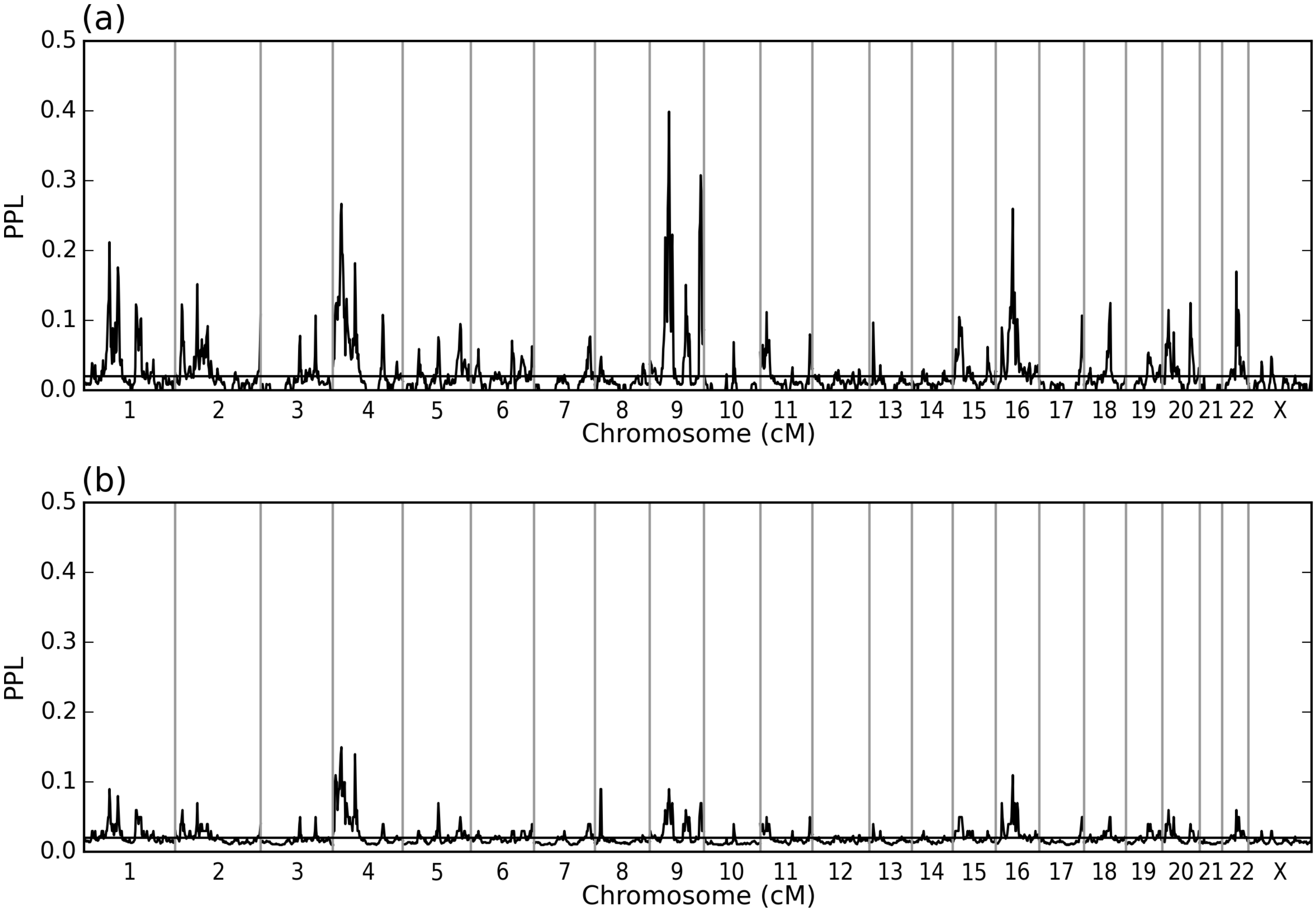

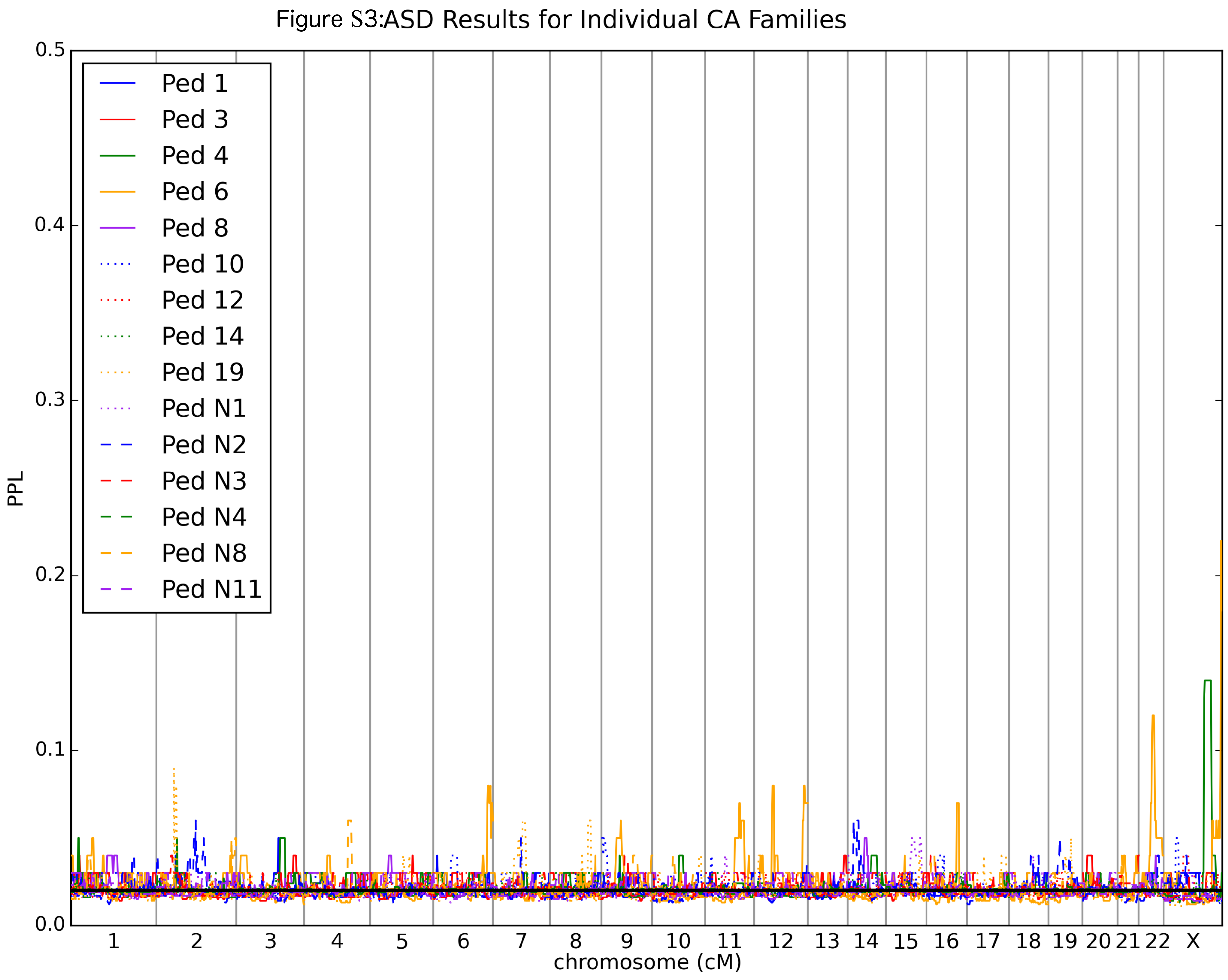
Figure S3:ASD Results for Individual CA Families
PPL
chromosome (cM)
0.0
0.1
0.2
0.3
0.4
0.5
1
2
3
4
5
6
7
8
9
10
11
12
13
14
15
16
17
18
19
20
21
22
X
Ped 1
Ped 3
Ped 4
Ped 6
Ped 8
Ped 10
Ped 12
Ped 14
Ped 19
Ped N1
Ped N2
Ped N3
Ped N4
Ped N8
Ped N11

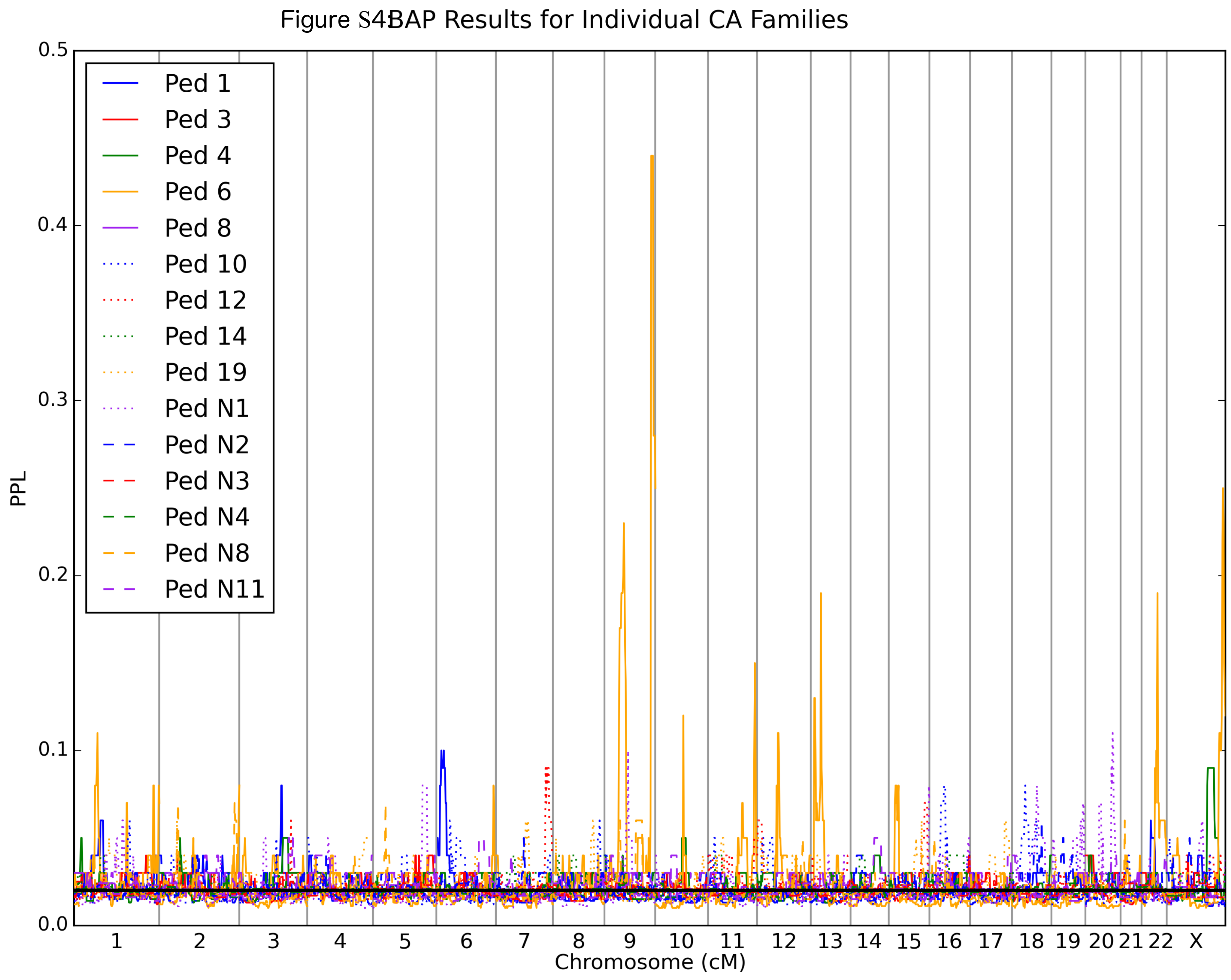
Figure S4B
BAP Results for Individual CA Families
PPL
Chromosome (cM)
0.0
0.1
0.2
0.3
0.4
0.5
1
2
3
4
5
6
7
8
9
10
11
12
13
14
15
16
17
18
19
20
21
22
X
Ped 1
Ped 3
Ped 4
Ped 6
Ped 8
Ped 10
Ped 12
Ped 14
Ped 19
Ped N1
Ped N2
Ped N3
Ped N4
Ped N8
Ped N11

Table S2: Salient ASD, BAP linkage peaks, Canadian and UNC pedigrees

Chr & Band	ASD PPL (%)	BAP PPL (%)	Peak (cM)[1]	Narrow[2]	Intermed	Broad	Peak (BP Position)[3]	ASD gene list[4] overlap (n=narrow, i=intermediate, b=broad)
1p36.22	**34%**	12	26	22-28	16-34	0-34	11957977	MTHFR (n, i, b), CAMTA1 (i,b), CA6 (i,b),
2q37.2	**25%**	16	250	250	246-250	244-256	236361323	AGAP1 (b)
6q27	**21%**	2	182	182	180-184	178-188	165645201	PDE10A (i,b), RPS6KA2 (b), RNASET2(b)
8q24.22	**27%**	14[4]	148	148-150	148-150	144-150	134467348	Nil
12p13.31	**29%**	1.5	20	20	18-22	10-28	7531425	C12orf57(i,b),
16p13.2	**33%**	4	24	16-26	10-28	8-28	9330226	RBFOX1 (n,i,b), ABAT (n,i,b), GRIN2A (n,i,b), CREBBP (b),
22q13.1	**45%**	1.5	50	46-62	44-62	42-64	37698639	CACNG2 (n,i,b), TNRC6B (n,i,b), ADSL (n,i,b), SGSM3 (n,i,b), EP300 (n,i,b)
2p13.1	3%	**22**	98	98	96-100	92-104	74913089	PER2(n,i,b), HDAC4 (n,i,b), KIF1A (n,i,b), D2HGDH (n,i,b), AGAP1 (i,b)
2q37.3	2%	**75**	264	252-264	248-264	246-264	243361159	Nil
9p21.3	1.6%	**67**	48	44-62	44-72	42-82	24428328	Nil
9q31.2	4%	**28**	112	112	110-116	102-118	109889954	ELAVL2 (n,i,b), LINGO2 (n,i,b), TAF1L (n,i,b), PAX5 (n,i,b), PIP5K1B (i,b), TRPM3 (i,b), ANXA1 (i,b), GNA14 (b)
15q13.3	6%	**62**	22	20-28	14-32	10-32	31770967	CYLC2 (b)
18q21.1	3%	**24**	72	72	70-72	70-82	45574928	TRPM1 (n,i,b), NSMCE3 (i,b), APBA2 (i,b), CHRNA7 (i,b), GABRB3 (b)
Xp22.11	2%	**21**	40	40	40-42	38-46	22295443	KATNAL2 (i,b), MBD1 (b), SMAD4 (b), TCF4 (b) RPS6KA3 (b), SMS (b), DDX53 (b), PTCHD1 (b)

[1]Both ASD and BAP PPLs are shown at the same location, corresponding to the location for the phenotype with the larger PPL (indicated in bold). [2]Peak width in cM, defined as the contiguous region around the peak for which the PPL remains ≥ 0.20 (Narrow), ≥ 0.10 (Intermediate) or ≥ 0.05 (Broad), for the phenotype with the larger PPL. [3]Physcial positions reference Build 37 and are included for convenience only; linkage analysis has resolution of approximately 1 cM (on average, around 1M basepairs) at best. [4]Under the BAP phenotype there is a PPL=23% at 150 cM, within the narrow range of the ASD peak. [4]ASD-gene list derived from Yuen et al. [Add: Yuen et al., 2017]
